# Substituting Fish Meal with Tubiechong (*Eupolyphaga sinensis*) By-Product in the Diets of Largemouth Bass (*Micropterus salmoides*): Effects on Growth, Meat Quality, and Liver Health

**DOI:** 10.1155/2023/2066602

**Published:** 2023-06-06

**Authors:** Yuhua Yue, Binbin Tong, Mingshi Chen, Xiaoxue Bao, Yanming Qiu, Ying Yang, Hui Yu, Yingying Yu

**Affiliations:** ^1^Guangdong Provincial Key Laboratory of Animal Molecular Design and Precise Breeding, School of Life Science and Engineering, Foshan University, Foshan, Guangdong 528225, China; ^2^Sinopharm Group Dezhong (Foshan) Pharmaceutical Co., Ltd., Foshan 528225, China

## Abstract

A feeding trial was conducted to evaluate the effect of replacing 0% (control), 10% (T10), 20% (T20), 30% (T30), and 40% (T40) fish meal with a Tubiechong (*Eupolyphaga sinensis*) by-product in largemouth bass (*Micropterus salmoides*). Triplicate groups of 30 fish (5.36 ± 0.01 g) were fed two times daily to apparent satiation for 60 days. The experimental results showed that the Tubiechong by-product could improve the growth performance of largemouth bass by increasing the FBW, WGR, and SGR until the replacement ratio was 40%. The quadratic regression analysis showed that the proportion of the Tubiechong by-product was 20.79% and 20.91%, respectively, when WGR and SGR were the best. Concurrently, the meat quality in the replacement groups was higher, specifically, the lightness and white values were higher, and the water loss rates were lower (*P* < 0.05) than that in the control group. Moreover, the changes of the activities of CAT and GSH in the liver and T-AOC and GSH in serum could reveal the antioxidant capacity improvement of fish by the Tubiechong by-product. In the study, the replacement groups had lower T-CHO and HDL-C in serum (*P* < 0.05), indicating that the Tubiechong by-product had an active role in improving blood lipid and regulating lipid metabolism. Simultaneously, the replacement groups had a normal structure with central hepatocytes' nuclei and deviated from the center partly, while most of the hepatocytes were swollen in the control group with nuclear degeneration. The results showed that the Tubiechong by-product had a positive effect on the liver health of fish. Conclusively, the present study indicated that the partial dietary replacement of fish meal using the Tubiechong by-product (for up to 40% replacement level) in the diet of largemouth bass not only caused no adverse effects on fish health but also improved the growth performance, meat quality, antioxidant capacity, and hepatic health and is conducive to supplying nutritious, high-quality, and healthy aquatic products.

## 1. Introduction

Largemouth bass is a typical carnivorous fish with a sky-high demand for dietary protein (mainly from fish meal), which has become an essential freshwater aquaculture species in China due to its strong adaptability, fast growth, and good meat quality [1, 2]. However, along with the speedy development of the aquaculture industry, global fish meal production could not meet the production needs of the feeding industry [3], and this situation had also forced people to find plant-based protein to replace fish meal. But many studies showed that the antinutritional substances in plants [4], such as phenolic and flavonoid compounds [5, 6], had adverse effects on feed intake, growth performance, and intestinal health. One study also found that completely replacing fish meal with a mixture of plant protein might not only affect sensory quality but also affect the nutritional composition [7]. Thus, to avoid the adverse effects of plant protein on the meat quality and nutritional value of fish, animal protein sources with high protein content and balanced distribution of essential amino acids had become the focus of research [8]. It was reported that animal protein sources replacing fish meal ranged from insects (mopane worms (*Imbrasia belina*), grasshoppers (*Zonocerus variegatus*), field crickets (*Gryllus bimaculatus*), blowfly maggot (*Chrysomya megacephala*), black soldier fly (*Hermetia illucens*), and superworm (*Zophobas morio*)), terrestrial animal by-products (fermented feather meal, feather meal, poultry by-products, meat and bone meal, and blood meal), fishery by-products (fish silage, shrimp head meal, and krill meal) [9], and animal traditional Chinese medicine, such as earthworm meal [10].

Tubiechong (*Eupolyphaga sinensis*), an animal traditional Chinese medicine, is rich in proteins, amino acids, peptides, fatty acids, alkaloids, nucleosides, polysaccharides, fat-soluble vitamins, and mineral elements [11]. According to the report, Tubiechong or its extract has many beneficial effects, including anticoagulation and antithrombosis [12], antioxidant [13], antitumor [14], immune regulation [15], blood lipid regulation, and hepatoprotection [16, 17]. Tubiechong by-product is the residence of Tubiechong after extracting the effective components. Nevertheless, the traditional processing could not obtain all active components [18], and the chemical composition results showed that the Tubiechong by-product still contained 70.36% crude protein. Therefore, it is highly probable that the Tubiechong by-product could partially replace fish meal as the protein source and may improve meat quality and nutritional value of aquatic animal through their own nutritional and bioactive properties. However, at present, neither the production value of the Tubiechong by-product for aquatic animal feed nor the optimal replacement level for improving aquatic animal meat quality has been studied.

To sum up, the study's objective is to assess the effect of the Tubiechong by-product as a partial replacement for fish meal on growth performance, serum biochemistry, liver health, and meat quality of largemouth bass, to explore the potential nutrition and health of the Tubiechong by-product and its effect on largemouth bass, and to provide a theoretical basis and practical reference for the sustainable and healthy development of high-quality aquatic products and aquaculture.

## 2. Materials and Methods

### 2.1. Diet Preparation

In the current study, five diets were formulated, as shown in Table 1 (% of dry matter). The control diet contained 100% of FM (T0), and the replacement groups replaced 10% (T10), 20% (T20), 30% (T30), and 40% (T40) fish meal by the Tubiechong by-product. In the diets, the protein was mainly provided by fish meal, soybean meal, peanut meal, and Tubiechong by-product, the primary lipid source was provided by fish oil, and wheat flour provided the carbohydrate source. Crystalline amino acids (lysine, methionine) also were added to the replacement groups (T10, T20, T30, and T40) to meet and balance dietary amino acid requirements. Thoroughly mix the raw materials of all dry substances, and add oil and water for mixing. Then, use a DS32-II twin-screw extruder (Guangzhou Vilavi Mechanical Equipment Co., Ltd.) to extrude the mixture (with a diameter of 1.5 mm), use a fan to air dry, and store it at -20°C until the start of the breeding experiment.

### 2.2. Feeding Trial and Experimental Conditions

A recirculating water system at Foshan University was used to breed experimental fish purchased from Guangdong Ho's Aquatic Products Co., Ltd. A total of 450 fish were randomly divided into 15 buckets (400 liters) after two weeks of acclimation. Three replica buckets (30 fish each) were used in every group. During the study, the experimental fish were fed at 8:00 and 17:30 for 60 days. The experimental period, water temperature, pH, NH4+, nitrite, nitrate, and dissolved O_2_ in water were maintained at 25 to 30°C, 6.5 to 7.5, <1 mg/L, <1 mg/L, <20 ppm, and >5.8 mg/L, respectively.

### 2.3. Measurement of Fish Growth Performance and Morphometric Parameters

The largemouth bass were made to fast for 24 h and then anaesthetized with buffered MS-222. A random sample of three fish was taken from each bucket, and their length and weight were recorded, respectively. After this, dissect and record their visceral and liver weights. The growth performance was calculated using the following formula: weight gain rate (WGR%) = (final body weight–initial body weight)/initial body weight × 100%, survival rate (SR%) = final fish number/initial fish number × 100%, specific growth rate (SGR%/day) = [ln (final body weight) − ln (initial body weight)]/days of feeding trial × 100%, feed conversion ratio (FCR) = Feed intake/weight gain, condition factor (CF g/cm^3^) = body weight/body length^3^ × 100%, viscerosomatic index (VSI%) = visceral weight/body weight × 100%, and hepatosomatic index (HSI%) = liver weight/body weight × 100%.

### 2.4. Determining Whole-Body and Muscle Chemical Composition

Three whole fish from each bucket and the dorsal muscles of the above 3 fish were sampled and stored at -20°C. The content of crude protein, crude lipid, crude ash, and moisture of the experimented diets, whole body, and muscle was determined by the Kjeldahl method, Soxhlet extraction, muffle furnace burning method (550°C, 4 h), and oven drying method (105°C, 6 h).

### 2.5. Enzyme Assays

Thirteen fish were randomly selected from each bucket, and the blood was collected from fish's caudal vein. The blood samples were centrifuged (2500 rpm/min, 15 min) at 4°C, and the supernatant (serum) was stored at -80°C until the determination of total cholesterol (T-CHO), triglyceride (TG), high-density lipoprotein cholesterol (HDL-C), total antioxidant capacity (T-AOC), and glutathione (GSH). Three fish were selected randomly from each bucket, and their livers were collected, made into liver homogenate, and centrifuged for 10 min (2500 rpm/min, 4°C); then, the supernatant was frozen at -80°C until the determination of superoxide dismutase (SOD), catalase (CAT), malondialdehyde (MDA), and glutathione (GSH). The determination of the above indicators was conducted under the guidance of the instructions of the kits of the Nanjing Jiancheng Bioengineering Institute.

### 2.6. Muscle Quality Measurement

Select 6 fish randomly from each bucket and collect their back muscles. Modify the previous research methods [19] to measure the pH, lightness (*L*^∗^), redness (*a*^∗^), yellowness (*b*^∗^), drop loss, thawing loss, cooking loss, and stored loss. Measure the muscle pH with a direct pH meter (accurate to 0.01, pH star, Mets, Germany), and measure the *L*^∗^, *a*^∗^, and *b*^∗^ with a colorimeter (SCQ-1A Tenovo International Co., Limited). The whiteness (*W*_*H*_) value was calculated according to the *L*^∗^, *a*^∗^, and *b*^∗^ values, and the formula was as follows:

(1) $$\begin{array}{c} W_{H}={\left[100-{\left(100–L^{*}\right)}^{2}+{\left(a^{*}\right)}^{2}+{\left(b^{*}\right)}^{2}\right]}^{1/2}. \end{array}$$

### 2.7. Histological Observations of Fish Liver

Select 2 fish randomly from each bucket, collect the livers carefully, fix them in 4% paraformaldehyde, dehydrate them in a graded alcohol series, clear them in xylol, and embed in paraffin, with the section at 5 *μ*m thickness, using hematoxylin and eosin (H&E) staining. Lastly, observed the stained sections under the microscope camera NLCD 500 (Nanjing, China).

### 2.8. Statistical Analyses

All data in the research were statistically analyzed using SPSS 26.0 (SPSS Inc., Michigan Avenue, Chicago, IL, USA). Use one-way ANOVA followed by Duncan's multiple-range tests. Quadratic polynomial was used to fit the correlation between WGR, SGR, and the proportion of the Tubiechong by-product replacing fish meal. All results were expressed as means ± S.E.M (standard error of the mean). Differences were considered as significant at *P* ≤ 0.05.

## 3. Results

### 3.1. Growth Performance and Morphometric Parameters

Table 2 shows the growth performance and morphometric parameters. Significantly (*P* < 0.05) higher FBW and WGR were reported for fish fed T20 diet compared to other diets. The SGR in the T20 group was significantly higher than that in the control and T40 groups (*P* < 0.05). The FCR in the control, T10, and T20 groups were significantly lower than that in T30 and T40 groups (*P* < 0.05). In addition, the CF and VSI did not differ among the different groups (*P* > 0.05), the HSI decreased gradually with the increase of the level of fish meal replaced by the Tubiechong by-product, and the HSI in T40 was significantly lower than that in the control and T10 groups (*P* < 0.05).

**Figure 1:**
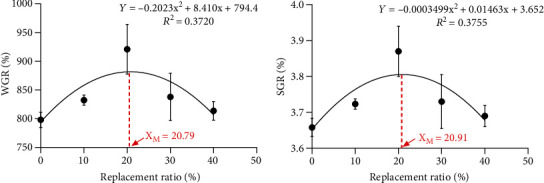
The relationship between WGR, SGR, and Tubiechong by-product replacement ratio of largemouth bass. (a) Weight growth rate (WGR); (b) specific growth rate (SGR).

Quadratic polynomial was used to fit the correlation between WGR, SGR, and the substitution ratio of the Tubiechong by-product (Figures 1(a) and 1(b)). According to the parabolic regression, the regression equations of WGR and SGR are *Y* = −0.2023*x*^2^ + 8.410*x* + 794.4 (*R*^2^ = 0.3720) and *Y* = −0.0003499*x*^2^ + 0.01463*x* + 3.652 (*R*^2^ = 0.3755), respectively. The maximum values of WGR and SGR of the largemouth bass were obtained at the highest point of the parabola, and the corresponding substitution ratios of the Tubiechong by-product were 20.79% and 20.91%, respectively. Therefore, the maximum replacement ratio of the Tubiechong by-product to meet the WGR and SGR of largemouth bass should be 20.78% to 20.91%.

### 3.2. Whole-Body Chemical Composition

Table 3 shows the whole-body chemical composition. Crude protein, crude ash, and moisture did not differ among the different groups (*P* > 0.05). The crude lipid content increased gradually with the increase of the level of the Tubiechong by-product, and the crude lipid in the T40 group was significantly higher than that in the T10 group (*P* < 0.05).

### 3.3. Meat Quality

Table 4 shows the results of meat quality. No difference was observed in either pH or *a*^∗^ value among the different groups (*P* > 0.05); the *L*^∗^, *b*^∗^, and *W*_*H*_ values in fish fed the T10 diet were higher than those in fish fed other diets (*P* < 0.05). Furthermore, compared with the control group, the drip loss in the replacement groups was lower (*P* < 0.05). The cooking loss in the control group was significantly higher than that in the T40 group (*P* < 0.05), and the stored loss was significantly higher than that in the T20, T30, and T40 groups (*P* < 0.05).

### 3.4. Analysis of Serum Biochemical Indices

**Figure 2:**
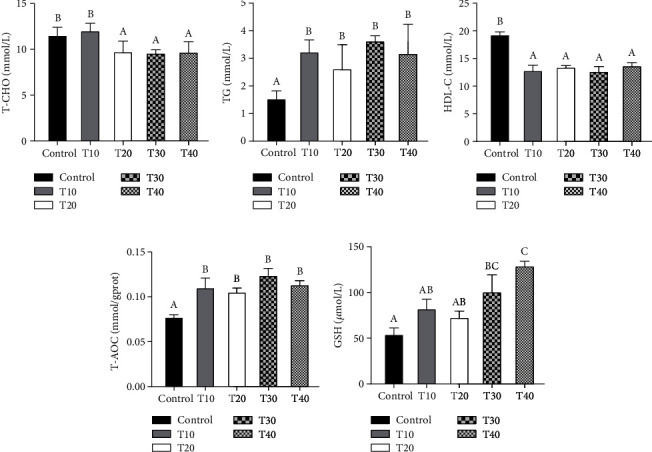
Effect of fish meal substituted with the Tubiechong by-product on serum biochemical indices of largemouth bass. (a) Total cholesterol (T-CHO); (b) triglyceride (TG); (c) high-density lipoprotein cholesterol (HDL-C); (d) total antioxidant capacity (T-AOC); (e) glutathione (GSH). Values marked with different letters are significantly different (*P* < 0.05) between treatments.

As illustrated in Figure 2, compared with the control and T10 groups, the serum T-CHO contents in the T20, T30, and T40 groups were significantly lower (*P* < 0.05) (Figure 2(a)). The TG contents in the replacement groups were significantly higher than the control group (Figure 2(b)); meanwhile, the HDL-C content was significantly lower than that in the control group (*P* < 0.05) (Figure 2(c)). Besides, the T-AOC activity in the control group was significantly lower than that in the replacement groups (*P* < 0.05) (Figure 2(d)), and the GSH content was significantly lower than that in the T30 and T40 groups (*P* < 0.05) (Figure 2(e)).

### 3.5. Analysis of Antioxidant Enzymes in the Liver

**Figure 3:**
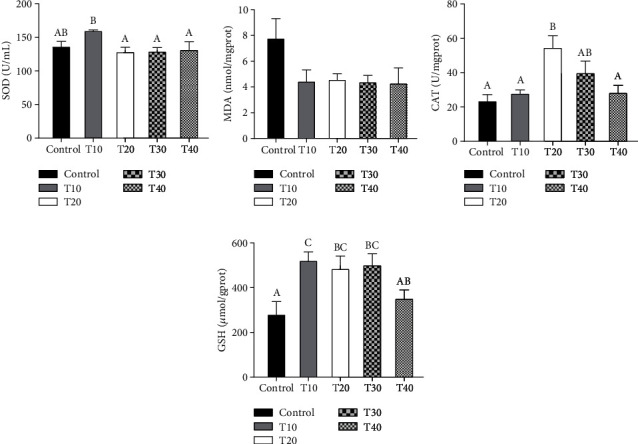
Effect of fish meal substituted with the Tubiechong by-product on liver antioxidant capacity of largemouth bass. (a) Superoxide dismutase (SOD); (b) malondialdehyde (MDA); (c) catalase (CAT); (d) glutathione (GSH). Values marked with different letters are significantly different (*P* < 0.05) between treatments.

As illustrated in Figure 3, the SOD activity in the T10 group was significantly higher than that in other replacement groups (*P* < 0.05) (Figure 3(a)), and the CAT activity in the T20 group was significantly higher than that in other groups except the T30 group (*P* < 0.05) (Figure 3(c)). The MDA contents in the replacement groups were lower than that in the control group (*P* > 0.05) (Figure 3(b)). The GSH contents in the T10, T20, and T30 groups were significantly higher compared with the control group (*P* < 0.05) (Figure 3(d)).

### 3.6. Liver Histology

**Figure 4:**
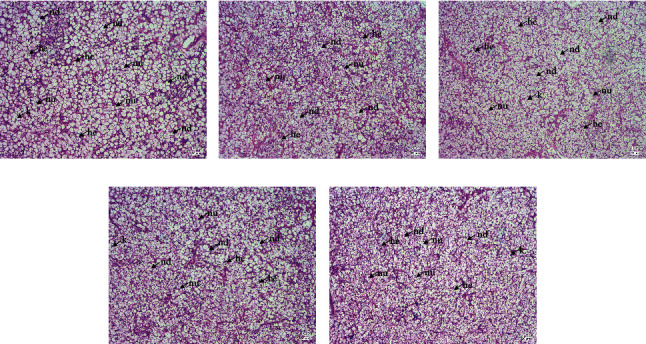
Effects of fish meal substituted with the Tubiechong by-product on liver histology of largemouth bass. he: hepatocytes; nu: nucleus; nd: nucleus disappear; k: karyolysis. Scale bar = 20 *μ*m. (a) Control group; (b) T10 group; (c) T20 group; (d) T30 group; (e) T40 group.

As illustrated in Figure 4, there was no apparent inflammatory cell infiltration in different groups. However, the irregular shape of hepatocytes in the control group was observed, mainly exhibiting signs of swollen hepatocyte cells and nuclear degeneration (such as nucleus disappearance and karyolysis), and large lipid droplets were detected (Figure 4(a)). In general, the hepatocytes of fish fed T10 diet had a regular structure, with most of the nuclei in the center and a few at the edge of the cells. Furthermore, a small amount of vacuolization of hepatocytes was observed (Figure 4(b)). Fish fed the T20, T30, and T40 diets showed a similar histomorphology to that in the T10 group; in contrast, a small number of large lipid droplets were detected in the T30 group (Figures 4(c)–4(e)).

### 3.7. Economic Benefits

Table 5 shows the economic benefits of the Tubiechong by-product instead of fish meal in culturing largemouth bass. When the largemouth bass gained 1000 kg in weight, compared with the control group, the Tubiechong by-product could save, respectively, 109.31, 569.36, 633.26, and 679.40 yuan when replacing 10%, 20%, 30%, and 40% of fish meal.

## 4. Discussion

In this study, partial replacement of fish meal with Tubiechong by-product not only had no adverse effect on growth performance and morphometric parameters of fish but also significantly increased FBW, WGR, and SGR when replacing 20% fish meal, which was similar to the results of previous studies. Tippayadara et al. found that there were no significant differences in growth indexes, feed utilization efficiency indices, feed intake, and survival rate of Nile tilapia (*Oreochromis niloticus*) between the fish meal group and black soldier fly (*Hermetia illucens*) substitute groups [20]. Chemello et al. also found that the growth and morphometric parameters of rainbow trout (*Oncorhynchus mykiss*) were not affected by defatted yellow mealworm (*Tenebrio molitor*) dietary inclusion [21]. Similarly, partially or entirely replacing fish meal with cricket (*Gryllus bimaculatus*) or superworm (*Zophobas morio*) did not lead to negative effects on the growth of olive flounder (*Paralichthys olivaceus*) or rainbow trout (*Oncorhynchus mykiss*), respectively [22, 23]. In the study, when the substitution level reached 40%, the growth performance decreased slightly. We speculated that it might be due to chitin, as the main component of some insect exoskeletons [24, 25] has high crystallinity and is completely insoluble in most organic and inorganic solvents [26]. So, it has a potential harmful effect on protein digestibility. Besides, it was reported that adult Tubiechong contained 11.50% chitin, which may result in growth performance decline [27]. In addition, the results of FCR supported this conjecture, which increased with the fish meal substitution level.

According to the reports, many factors could affect the nutritional composition of fish, such as genetic factors [28], water environment [29, 30], and season [31], and the feed nutrition is the most critical factor [32]. In this study, there was no significant effect of fish meal substituted with the Tubiechong by-product on crude protein, crude ash, and moisture of largemouth bass. However, the crude lipid in the T40 group was significantly higher than that in the T10 group. We conjectured that due to the adverse effect of chitin in the Tubiechong by-product, the digestive capacity of largemouth bass decreases, resulting in higher lipid content of fish [33]. Therefore, the crude lipid content increased progressively with the increase of the level of fish meal replaced by the Tubiechong by-product. Similar results were shown in previous studies [22, 34]. The above results indicated that partial replacement of fish meal by the Tubiechong by-product positively affected the growth performance of largemouth bass to some extent.

In general, the research showed that replacing fish with Tubiechong by-product could improve the growth performance of bigmouth bass, which might be related to the nutritional composition of the Tubiechong by-product. Odunayo et al. proposed that insects were rich in protein representing the main component of their nutrient composition, and they also contained an abundance of other significant nutrients such as lipids, beneficial fatty acids, vitamins, and minerals, which could improve the growth performance of animal [35–38]. Therefore, we speculated that the Tubiechong by-product with 70.36% crude protein and 15.95% crude lipid also contained the above nutrients, and the largemouth bass had a higher degree of absorption and utilization of feed, which was consistent with the FCR results in the study. Besides, the largemouth bass might prefer the fishy taste of the Tubiechong by-product and have a higher acceptance of feed, which might also be related to the improvement of growth performance.

For producers and consumers, meat quality is an essential characteristic. Meat quality is not only related to sensory properties (color, juiciness, and flavor) [39] but also closely related to its physical and chemical parameters, such as water holding capacity (WHC), pH, and nutrients [40]. The pH is one of the most critical factors affecting meat color, tenderness, WHC, and other characteristics of muscle [41], and the tenderness of fish meat decreases with the pH decrease. As one of the critical indicators to evaluate the freshness of fish, fish color has a significant impact on consumers' purchase decisions [42]. In our current study, the *L*^∗^, *b*^∗^, and *W*_*H*_ values in the T10 group were significantly higher than those in other groups, indicating that the Tubiechong by-product could improve the body color of largemouth bass and make consumers more eager to buy. Moreover, it was reported that the stress degree of fish before death was lower, and the *L*^∗^ value of fish fillet was lower [43, 44], which indicated largemouth bass fed with the Tubiechong by-product were less stressed.

Moreover, WHC is of great essentiality to muscle's physical form, flavor, and color, which can be appraised using drip loss, thawing loss, cooking loss, and stored loss. In the study, the drip loss in the replacement groups, the cooking loss in the T40 group, and the stored loss in the T20, T30, and T40 groups were lower significantly compared with the control group. This showed that the Tubiechong by-product could enhance the WHC of largemouth bass muscle to improve its meat quality. A previous study reported a negative correlation between WHC and MDA contents in muscle [45]. In this research, the liver MDA contents in the replacement groups were lower than that in the control group. In addition, the study also showed that the WHC of muscle was closely related to pH value, and when the pH value decreased, WHC decreased [46]. In this research, the pH values in the replacement groups were lower compared with the control group. Therefore, the Tubiechong by-product could enhance the WHC by reducing the oxidative damage and increasing the pH value possibly. Generally, the Tubiechong by-product positively impacted meat color, pH, and WHC, resulting in higher nutritional value, better appearance, and palatability of largemouth bass.

Antioxidant enzymes are the primary protective mechanism of protecting fish tissues from oxidative stress [47]. The SOD and CAT can protect the organism against damnification by reactive oxygen species (ROS), which causes many disorders [48]. As the most crucial nonenzymatic antioxidant in fish, GSH has the functions of scavenging free radicals and detoxifying [49]. MDA is an essential product of lipid peroxidation, which can interact with free amino acids of proteins to cause body cell damage [50], and the degree of oxidative damage in the liver and body cells can be reflected by MDA content [51]. In this experiment, SOD and CAT reached peak activity levels in the T10 and T20 groups, respectively. Concurrently, the GSH content was higher in the T10, T20, and T30 groups than that in the control group, and the contents of MDA showed no differences among all groups. Similarly, Abdel-Latif et al. and Caimi et al. found that black soldier fly (*Hermetia illucens*) substitution for fish meal could increase SOD and CAT activities of Siberian sturgeon (*Acipenser baerii*) and European seabass, respectively [52, 53]. The above results indicated that the Tubiechong by-product had a positive effect on the antioxidant capacity of largemouth bass, which was achieved by increasing CAT activity and GSH content and decreasing MDA content possibly.

In addition, total antioxidant capacity (T-AOC), as a comprehensive indicator of antioxidant capacity, reflects the cumulative effects of all antioxidants in plasma and body fluids [54]. GSH plays a vital role in antioxidation and liver detoxification [55]. In the study, the serum T-AOC and GSH contents in the replacement groups were higher than those in the control group, indicating that the Tubiechong by-product could improve the antioxidant capacity of largemouth bass by increasing T-AOC and GSH content. Similar results were also reported that defatted black soldier fly (*Hermetia illucens*) larva meal or yellow mealworm (*Tenebrio molitor*) substitution for fish meal could increase T-AOC and GSH contents of Chinese soft-shelled turtle (*Pelodiscus sinensis*) or largemouth bass (*Micropterus salmoides*), respectively [47, 56]. Besides, there is a positive correlation between meat quality and muscle antioxidant capacity. So, we speculated that the improvement in meat quality in the replacement groups might be attributed to the increase in antioxidant capacity.

Serum biochemical indexes can reflect the health and physiological status of fish. Total cholesterol (T-CHO) and triglyceride (TG) in serum reflect the ability of liver fat metabolism and lipid absorption fat metabolism in the liver to some extent [57, 58]. HDLC can transfer cholesterol from extrahepatic tissue to the liver for metabolism [58]. Wang et al. found that the active peptide AR-9 extracted from Tubiechong could reduce blood lipid by reducing the T-CHO, TG, and HDL-C levels, which was consistent with the changes of T-CHO and HDL-C in this study [59]. In the study, the T-CHO contents in the replacement groups were lower compared with the control group, and the HDLC content showed a similar trend, which indicated that more T-CHO in serum was transferred to the liver by HDLC. The Tubiechong by-product could reduce blood fat by lowering the T-CHO content. In addition, in this study, the whole-body lipid content decreased with the decrease in plasma triglyceride concentration, which was consistent with the previous research [60]. Shen et al. used cottonseed protein concentrate to replace fish meal and found that the T-CHO and HDL-C contents decreased in the serum of golden pompano (*Trachinotus ovatus*) [61], and Hossain and Koshio found similar results [62]. These results were consistent with this study. Thus, the above results showed that the Tubiechong by-product positively impacted lipid metabolism, improved body health of largemouth bass, and then provides high-quality aquatic product.

As the central metabolic organ, the liver of fish controls both growth and nutritional characteristics, including the quality and quantity of fish fillets [2]. Therefore, the histological changes of the liver can reflect the nutrition-related pathological changes of fish [63]. In the study, the hepatocytes of largemouth bass fed control diet showed swelling and nuclear degeneration, and large lipid droplets were observed simultaneously. The structure of hepatocytes in the T10 and T20 groups was regular, and most of the nuclei were located in the center. These results indicated that the Tubiechong by-product could ameliorate the liver lipidosis of largemouth bass, and the significant decrease in the contents of serum T-CHO and HDL-C might further verify this prediction. Besides, Xie et al. proposed that the Tubiechong by-product could alleviate hepatic fibrosis, which was dependent on its peptides [11], while deeper mechanisms required further study. Substantially, the Tubiechong by-product could protect the liver and further improve the health and nutritional value of largemouth bass.

## 5. Conclusion

Based on the results, replacing dietary fish meal with different levels of Tubiechong by-product could improve the nutritional value and meat quality of largemouth bass by enhancing its growth performance, meat color, water-holding capacity, and antioxidant capacity; improving liver health; and saving certain feed costs. According to the binary polynomial regression analysis, the appropriate proportion of Tubiechong by-product instead of fish meal in largemouth bass feed should be 20.79%-20.91%.

## Figures and Tables

**Table 1:** Composition and proximate analysis of the five experimental diets (% of dry matter).

Ingredient	Groups				
	Control	T10	T20	T30	T40
Tubiechong by-product^1^	0.00	4.50	9.00	13.50	18.00
Fish meal^2^	45.00	40.50	36.00	31.50	27.00
Fermented soybean meal^3^	15.00	15.00	15.00	15.00	15.00
Peanut meal	10.00	10.00	10.00	10.00	10.00
Wheat flour	11.00	10.78	10.57	10.34	10.12
Wheat gluten	7.00	7.00	7.00	7.00	7.00
Brewer's yeast	3.00	3.00	3.00	3.00	3.00
Soy lecithin	1.00	1.00	1.00	1.00	1.00
Fish oil	3.00	3.00	3.00	3.00	3.00
Choline chloride	0.50	0.50	0.50	0.50	0.50
Monocalcium phosphate	1.50	1.50	1.50	1.50	1.50
Premix^4^	3.00	3.00	3.00	3.00	3.00
Crystalline lysine	0.00	0.15	0.29	0.44	0.59
Crystalline methionine	0.00	0.07	0.14	0.22	0.29
Total	100.00	100.00	100.00	100.00	100.00
Proximate composition					
Moisture	8.25	8.59	7.96	7.86	8.39
Crude protein	49.24	49.64	50.19	50.31	50.83
Crude lipid	13.18	13.09	12.88	12.51	12.21
Crude ash	13.21	12.83	12.86	12.56	12.24

^1^Tubiechong by-product: crude protein, 70.36%; crude lipid, 15.95%; crude ash, 10.04%; moisture, 7.76%. ^2^Fish meal (Peru): crude protein, 66.07%; crude lipid, 11.82%. ^3^Fermented soybean meal provided by Yuanyao Biological Feed Co., Ltd., Shanghai, China. ^4^Vitamin mixture (kg^−1^ of diet): vitamin A, 250,000 IU; riboflavin, 750 mg; pyridoxine HCL, 400 mg; cyanocobalamin, 1 mg; thiamin, 250 mg; menadione, 250 mg; folic acid, 125 mg; biotin, 10 mg; *α*-tocopherol, 2.5 g; myoinositol, 8000 mg; calcium pantothenate, 1250 mg; nicotinic acid, 2000 mg; choline chloride, 8000 mg; vitamin D3, 45,000 IU; vitamin C, 7000 mg. Mineral mix (kg^−1^ of diet): ZnSO_4_·7H_2_O, 0.04 g; CaCO_3_, 37.9 g; KCl, 5.3 g; KI, 0.04 g; NaCl, 2.6 g; CuSO_4_·5H_2_O, 0.02 g; CoSO_4_·7H_2_O, 0.02 g; FeSO_4_·7H_2_O, 0.9 g; MnSO_4_·H_2_O, 0.03 g; MgSO_4_·7H_2_O, 3.5 g; Ca(HPO_4_)_2_·2H_2_O, 9.8 g.

**Table 2:** Effect of fish meal substituted with the Tubiechong by-product on growth performance and morphometric parameters of largemouth bass.

Items	Groups				
	Control	T10	T20	T30	T40
IBW (g)	5.35 ± 0.01	5.36 ± 0.01	5.35 ± 0.01	5.35 ± 0.01	5.36 ± 0.00
FBW (g)	48.09 ± 0.74^a^	50.01 ± 0.40^a^	54.61 ± 2.28^b^	50.22 ± 2.21^a^	48.94 ± 0.91^a^
SR (%)	100.00 ± 0.00^b^	100.00 ± 0.00^b^	100.00 ± 0.00^b^	97.78 ± 1.11^a^	100.00 ± 0.00^b^
WGR (%)	798.22 ± 13.34^a^	832.34 ± 8.82^a^	920.97 ± 43.43^b^	838.07 ± 41.15^a^	813.75 ± 16.29^a^
SGR (%)	3.66 ± 0.03^a^	3.72 ± 0.01^ab^	3.87 ± 0.07^b^	3.73 ± 0.08^ab^	3.69 ± 0.03^a^
FCR	0.82 ± 0.01^a^	0.85 ± 0.01^a^	0.84 ± 0.01^a^	0.89 ± 0.01^b^	0.93 ± 0.01^c^
CF (g/cm^3^)	1.36 ± 0.02	1.35 ± 0.03	1.39 ± 0.03	1.34 ± 0.02	1.35 ± 0.04
VSI (%)	8.24 ± 0.23	8.30 ± 0.36	8.19 ± 0.23	7.92 ± 0.19	7.92 ± 0.29
HSI (%)	3.41 ± 0.17^b^	3.39 ± 0.25^b^	3.21 ± 0.17^ab^	3.14 ± 0.21^ab^	2.67 ± 0.18^a^

Values marked with different letters are significantly different (*P* < 0.05) between treatments.

**Table 3:** Effect of fish meal substituted with the Tubiechong by-product on whole-body chemical composition of largemouth bass.

Items	Groups				
	Control	T10	T20	T30	T40
Crude protein	62.53 ± 0.08	63.58 ± 0.26	62.58 ± 0.91	62.22 ± 0.17	62.00 ± 0.65
Crude lipid	21.46 ± 0.47^ab^	21.24 ± 0.67^a^	21.94 ± 0.84^ab^	22.06 ± 0.67^ab^	23.38 ± 0.41^b^
Crude ash	12.95 ± 0.11	13.17 ± 0.14	13.15 ± 0.06	13.29 ± 0.09	13.06 ± 0.07
Moisture	3.14 ± 0.28	3.04 ± 0.33	3.55 ± 0.24	2.76 ± 0.15	3.18 ± 0.21

Values marked with different letters are significantly different (*P* < 0.05) between treatments.

**Table 4:** Effect of fish meal substituted with the Tubiechong by-product on meat quality of largemouth bass.

Items	Groups				
	Control	T10	T20	T30	T40
pH	6.33 ± 0.05	6.40 ± 0.04	6.45 ± 0.04	6.38 ± 0.04	6.35 ± 0.05
*L* ^∗^	48.73 ± 0.84^a^	51.70 ± 0.58^b^	49.19 ± 0.77^a^	48.78 ± 0.63^a^	47.56 ± 0.94^a^
*a* ^∗^	2.01 ± 0.43	2.43 ± 0.57	1.68 ± 0.54	1.82 ± 0.36	1.65 ± 0.23
*b* ^∗^	3.97 ± 0.60^a^	5.64 ± 0.36^b^	3.44 ± 0.62^a^	3.86 ± 0.36^a^	3.07 ± 0.21^a^
*W*_*H*_	48.52 ± 0.80^a^	51.35 ± 0.60^b^	49.01 ± 0.76^a^	48.59 ± 0.63^a^	47.42 ± 0.94^a^
Drip loss	3.68 ± 0.25^b^	2.78 ± 0.19^a^	2.80 ± 0.27^a^	2.57 ± 0.23^a^	2.91 ± 0.34^a^
Thawing loss	3.90 ± 0.61	3.13 ± 0.65	3.46 ± 0.49	2.38 ± 0.26	2.46 ± 0.33
Cooking loss	33.32 ± 1.67^b^	29.77 ± 0.70^ab^	30.23 ± 1.42^ab^	29.72 ± 0.84^ab^	29.18 ± 0.25^a^
Stored loss	4.04 ± 0.34^c^	3.32 ± 0.26^bc^	2.68 ± 0.18^ab^	2.18 ± 0.25^a^	2.58 ± 0.27^ab^

Values marked with different letters are significantly different (*P* < 0.05) between treatments.

**Table 5:** Economic benefits of the Tubiechong by-product instead of fish meal in culturing largemouth bass.

Items	Groups				
	Control	T10	T20	T30	T40
Feed price (yuan/t)	9976.08	9497.77	9019.47	8541.16	8062.85
Weight gain	3.85	4.02	4.42	4.07	3.91
Food intake	3.17	3.43	3.75	3.61	3.65
Consumption of feed cost (yuan)	31.58	32.55	33.80	30.83	29.46
Feed cost for each increased 1 kg body weight consumption (yuan)	8.21	8.10	7.64	7.58	7.53
Feed cost consumed by 1000 kg fish (yuan)	8211.76	8102.45	7642.40	7578.50	7532.36
Cost savings of 1000 kg fish (yuan)	0.00	109.31	569.36	633.26	679.40

## Data Availability

The data that support the findings of this study are available from the first author upon reasonable request.
